# High Prevalence of Tuberculosis and Serious Bloodstream Infections in Ambulatory Individuals Presenting for Antiretroviral Therapy in Malawi

**DOI:** 10.1371/journal.pone.0039347

**Published:** 2012-06-22

**Authors:** Richard A. Bedell, Suzanne T. B. Anderson, Monique van Lettow, Ann Åkesson, Elizabeth L. Corbett, Moses Kumwenda, Adrienne K. Chan, Robert S. Heyderman, Rony Zachariah, Anthony D. Harries, Andrew R. Ramsay

**Affiliations:** 1 Dignitas International, Zomba, Malawi; 2 Division of Global Health, University of British Columbia, Vancouver, British Columbia, Canada; 3 Malawi-Liverpool-Wellcome Trust Clinical Research Programme, Blantyre, Malawi; 4 Department of Medicine, Brighton and Sussex Medical School, Falmer, Sussex, United Kingdom; 5 Dalla Lana School of Public Health, University of Toronto, Toronto, Ontario, Canada; 6 Médecins Sans Frontières – Operational Centre Brussels, Thyolo, Malawi; 7 London School of Hygiene and Tropical Medicine, London, United Kingdom; 8 Department of Medicine, St. Michael's Hospital, University of Toronto, Toronto, Ontario, Canada; 9 Médecins Sans Frontières – Operational Centre Brussels, Brussels, Belgium; 10 International Union Against Tuberculosis and Lung Disease, Paris, France; 11 World Health Organization, Geneva, Switzerland; 12 School of Medicine, University of St. Andrews, Fife, Scotland; National Institute of Allergy and Infectious Diseases, United States of America

## Abstract

**Background:**

Tuberculosis (TB) and serious bloodstream infections (BSI) may contribute to the high early mortality observed among patients qualifying for antiretroviral therapy (ART) with unexplained weight loss, chronic fever or chronic diarrhea.

**Methods and Findings:**

A prospective cohort study determined the prevalence of undiagnosed TB or BSI among ambulatory HIV-infected adults with unexplained weight loss and/or chronic fever, or diarrhea in two routine program settings in Malawi. Subjects with positive expectorated sputum smears for AFB were excluded. Investigations Bacterial and mycobacterial blood cultures, cryptococcal antigen test (CrAg), induced sputum (IS) for TB microscopy and solid culture, full blood count and CD4 lymphocyte count. Among 469 subjects, 52 (11%) had microbiological evidence of TB; 50 (11%) had a positive (non-TB) blood culture and/or positive CrAg. Sixty-five additional TB cases were diagnosed on clinical and radiological grounds. Nontyphoidal *Salmonellae* (NTS) were the most common blood culture pathogens (29 cases; 6% of participants and 52% of bloodstream isolates). Multivariate analysis of baseline clinical and hematological characteristics found significant independent associations between oral candidiasis or lymphadenopathy and TB, marked CD4 lymphopenia and NTS infection, and severe anemia and either infection, but low positive likelihood ratios (<2 for all combinations).

**Conclusions:**

We observed a high prevalence of TB and serious BSI, particularly NTS, in a program cohort of chronically ill HIV-infected outpatients. Baseline clinical and hematological characteristics were inadequate predictors of infection. HIV clinics need better rapid screening tools for TB and BSI. Clinical trials to evaluate empiric TB or NTS treatment are required in similar populations.

## Introduction

Mortality among patients initiating antiretroviral therapy (ART) in low-income settings is greater than in high-income settings, particularly within the first 3–12 months of treatment [1]–[3]. This is especially true among patients presenting with unexplained weight loss, chronic fever or chronic diarrhea [2], [4]–[6].

Weight loss and fever among HIV-infected persons often reflect active tuberculosis (TB) and other serious bloodstream infections (BSI) [7]–[9]. Smear microscopy, the mainstay of diagnosis in these settings, is highly specific, but has low sensitivity especially in HIV-infected individuals [10], [11]. Several studies have evaluated the predictive value of various symptoms for the detection of TB [11]–[15], with cough, weight loss, fever, and night sweats each associated with active TB but with low specificity for culture-confirmed TB.

Studies performed in a variety of sub-Saharan African settings have shown that the most common BSI among HIV-infected persons hospitalised with acute or chronic fever are *Mycobacterium tuberculosis*, *Cryptococcus neoformans*, *Streptococcus pneumoniae*, nontyphoidal *Salmonella* (NTS) species, malaria and *Staphylococcus aureus* [7]–[9], [16]–[18]. These studies selected febrile patients who were ill enough to be admitted to hospital but it is not clear if their findings can be generalized to a larger population of ambulatory patients with a variety of non-specific symptoms of chronic illness. Screening of asymptomatic patients for cryptococcal infection have also demonstrated its presence among ambulatory HIV-infected patients [16], [17]. While several autopsy studies have identified specific causes of death among HIV-infected persons [19]–[22], they provide limited information about how to identify patients at risk of TB or other infections.

In this study, our primary aim was to prospectively determine the prevalence of TB and serious BSI among sputum smear negative, HIV-infected persons presenting as out-patients in a program setting with unexplained chronic fever and/or weight loss and/or chronic diarrhea. Our second aim was to determine if baseline clinical or hematological characteristics of this population were associated with identification of a pathogen.

## Methods

### Study Design

This was a prospective observational cohort study. Subjects were enrolled between February and November 2010.

### Ethics

This study received prior ethical approval from the National Health Sciences Research Committee of Malawi and from the Ethics Advisory Group of the International Union Against Tuberculosis and Lung Disease (IUATLD). Eligible patients provided written informed consent.

### Study Population

HIV-infected adults (≥15 years of age) undergoing assessment of eligibility for first-time initiation of antiretroviral therapy, or seeking health care, in outpatient clinics at Zomba Central Hospital and Thyolo District Hospital, serving 2 districts in the southern region of Malawi with high HIV co-infection rates of ≥70% among TB patients and adult HIV prevalence of 12%. Patients were eligible for inclusion in the study if they had three negative expectorated sputum smears (or if they were unable to expectorate) and at least one of the following criteria: history of unexplained severe weight loss, defined as >10% of baseline body weight (estimated or documented); or unexplained chronic fever (intermittent or continuous for >1 month duration); or unexplained chronic diarrhea (loose or watery stools three or more times daily) for >1 month duration; or unexplained moderate weight loss, defined as <10% of baseline body weight (estimated or documented) with CD4 count <250 cells/µL. Anti-TB therapy in the past month or pregnancy were exclusion criteria.

### Enrolment

**Figure 1:**
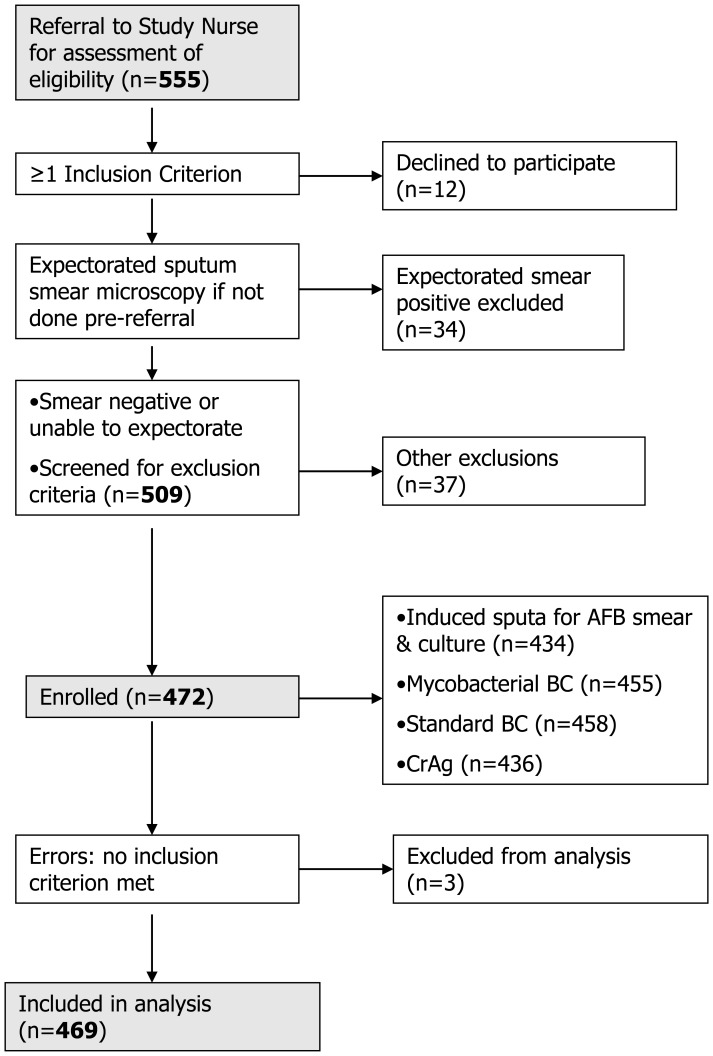
Study Enrolment Flowchart.

Clinical officers involved in routine clinical care identified potential participants and referred them to on-site study nurses. Nurses were involved in study-related diagnostic procedures only but not in clinical care. Diagnosis and treatment was provided by non-study clinicians. (See Figure 1).

### Procedures

A baseline interview included questions about demographics, clinical history and current symptoms. Weight and height were recorded, and venepuncture was performed.

Patients able to expectorate had sputum smear microscopy either prior to referral to a study nurse, or during the screening process. In either case, collection and examination of expectorated sputum was performed by non-study program personnel at the two enrolment sites. Malawi National TB Program guidelines stipulate one spot sputum specimen, one early morning specimen and another spot specimen when the morning specimen is submitted.

Sputum induction was carried out using nebulised, hypertonic (5%) saline under recommended biosafety conditions [23], [24] in an open-air location 100 metres away from other patients or staff; study nurses wore N95 respirators during the procedure. If sputum induction failed, participants were enrolled without a sputum specimen. A chest radiograph was obtained if not already available.

Participants were then managed under the routine care system at each hospital, but with the benefit of results provided by the study team. Information on clinical TB diagnoses made after enrolment and information on mortality was obtained from routine program (non-study) sources, although the study employed tracers to obtain some mortality data. Mortality and other outcomes are the subject of a separate analysis and paper. The enrolment process is shown in Figure 1.

### Laboratory Methods & Procedures

TB microscopy and cultures were performed at the TB laboratory of the College of Medicine/Malawi-Liverpool-Wellcome Trust Clinical Research Programme (MLW), Blantyre. This laboratory provides TB diagnostic support to clinical trials funded by the National Institutes of Health, Division of AIDS (NIH/DAIDS)/AIDS Clinical Trials Group (ACTG) and as such employs the quality management system recommended by the NIH, including an annual audit. The laboratory takes part in the External Quality Assurance programs of both the NIH and the UK National External Quality Assurance Scheme (NEQAS).

After same-day transport to the laboratory, induced sputum (IS) was decontaminated with an equal volume of 4% NaOH for 15 minutes and concentrated with centrifugation before culture onto Lowenstein-Jensen (LJ) media for up to eight weeks. Smears made from both direct and concentrated sputum were examined under fluorescent microscopy (Auramine O), with any positive results confirmed by Ziehl-Neelsen staining (ZN). Mycobacterial isolates were further speciated as *Mycobacterium tuberculosis* (MTB*)* or nontuberculous mycobacteria (NTM) using microscopic cording and MBP-64 lateral flow assays (Capilia®; TAUNS Laboratories, Inc., Numazu, Japan) and, if either test was negative, growth on media with p-nitrobenzoic acid (PNB) at room temperature and/or 45 deg C.

Blood for culture of mycobacteria and other bacterial (or fungal) pathogens was collected using a sterile technique and transferred the same day to the Microbiology Laboratory at Queen Elizabeth Hospital, Blantyre (supported by the Malawi-Liverpool-Wellcome Trust Clinical Research Programme). For bacterial culture, venous blood (5–7.5 mL) was taken for aerobic culture in 50 mL of broth and incubated for 7 days (BacT/ALERT 3D®; bioMérieux SA, Marcy l’Etoile, France). Isolates were then identified using standard diagnostic techniques [25]. For the purpose of this study, all organisms which are found as normal skin or oral flora were considered to be contaminants, including coagulase-negative *Staphylococci*, alpha-hemolytic *Streptococci* other than *S. pneumoniae,* and diphtheroids. Antibiotic susceptibility was determined by disc testing (Oxoid, Cambridge, UK) [26].

For mycobacterial culture, venous blood (5 mL) was inoculated into 50 mL broth (BACTEC Myco/F Lytic®; Becton Dickinson Microbiology Systems, Sparks, MD, USA) and incubated at 37 deg C. Bottles were inspected daily for the first 14 days and then once every 2 days using a handheld UV Woods lamp. Contents of bottles were concentrated by centrifugation (3000 g for 15 minutes) either within 48 hours after first detection of fluorescence, or at the end of 6 weeks incubation (whichever occurred sooner). The concentrate was examined with ZN and Gram’s staining to exclude bacterial contaminants, and subcultured onto LJ media. ZN positive subcultures were then speciated as above.

Cryptococcal antigen tests were conducted on a single 1∶10 dilution of serum using a *Cryptococcus* rapid latex agglutination test (Oxoid, Cambridge, UK).

CD4 counts were conducted on venous blood (CyFlow®; Partec GmbH, Goerlitz, Germany), as were full blood counts (AutoRead Plus®; QBC Diagnostics, Port Matilda, PA, USA).

### Definitions of Tuberculosis and Blood Stream Infections

TB was defined as confirmed, probable or possible using the following case-definitions: *Confirmed TB*: one or more colonies of MTB on culture of IS or blood. *Probable TB*: one or more acid-fast bacilli on smear microscopy from IS despite negative cultures if smears were confirmed as ZN positive on review by 3 independent readers. *Possible TB*: compatible clinical and/or radiographic findings with decision to treat for TB made by treating clinical officers; includes enrolled subjects who started TB treatment up to 3 months after study enrolment (confirmed through TB registers).

NTM disease was defined as *disseminated NTM disease* if there was isolation from blood of nontuberculous mycobacteria (NTM). The diagnosis of NTM lung disease was not possible in this study because only one sputum culture was taken per participant. Sputum NTM isolates were therefore of uncertain clinical significance. [27] We had no means for further speciation of NTM isolates.

BSI were defined as isolation of pathogenic bacteria or fungal organism (non-pathogenic species as defined in Methods were excluded) or a positive serum cryptococcal antigen titre of ≥1∶10 (in general a titre of ≥1∶8 is considered indicative of disseminated cryptococcal infection but the assay used in this study provided only a single titre of 1∶10; positives at this dilution were considered cases but higher dilutions of serum were not tested).

### Statistical Analysis

Data and statistical analysis were conducted using IBM SPSS Statistics 19 (IBM, Armonk, NY, USA). Baseline characteristics were described with proportions or medians (interquartile ranges [IQR]). Comparisons between groups were made using non-parametric independent sample median tests. Poverty score (using the household poverty assessment model for Malawi as described by Payongayong et al. [28]) was categorized into quartiles. Body Mass Index (BMI), CD4, Hb, WBC and platelets were categorized using conventional cut-off values. Multivariate logistic regressions models were fitted with “confirmed or probable TB” or “NTS” as the outcome variable. A minimum of 7 events per variable was required to include a predictor variable in a regression model. Adjusted odds ratios (aORs) (95% CI) were calculated for each model and were controlled for sex, age, poverty score, chronic fever (present >1 month; intermittent or continuous), weight loss >10%, chronic diarrhea (present 1 month or more), BMI, chronic cough (2 weeks or more), presence of oral candidiasis, lymphadenopathy, CD4 count, Hb, WBC count and platelets. Positive likelihood ratio was defined as [sensitivity/(1– specificity)]; negative likelihood ratio was defined as [(1– sensitivity)/specificity]. A significance level of 0.05 was set for all statistical testing.

## Results

### Patient Characteristics

There were 469 subjects (279 females) enrolled in the study and eligible for analysis, from February to December 2010, 285 at Zomba Central Hospital and 184 at Thyolo District Hospital. (See Table 1).

**Table 1:** Enrolment characteristics of 469 study subjects.

Characteristic	N (%) or Median (IQR)
Sex F	279 (58%)
Age (median; IQR)	36 (24–48)
Below national poverty line	233 (49.7%)
Fever, chronic*	294 (62.7%)
Weight loss, severe*(>10% body wt)	249 (53.1%)
Weight loss, moderate*(<10% body wt)	209 (44.6%)
Diarrhea, chronic*	165 (35.2%)
More than one symptom defined as inclusion criteria**	320 (68.2%)
BMI, kg/m^2^ (median; IQR)	17.1 (15.9–18.4)
Cough, chronic	284 (60.6%)
Oral Candidiasis	160 (34.1%)
Lymphadenopathy (any)	180 (38.4%)
CD4, cells/µL (median; IQR)	129 (49–216)
Hemoglobin, male, g/L (median; IQR)	100 (84–115)
Hemoglobin, female, g/L (median; IQR)	100 (82–115)
WBC, X10^9^/L (median; IQR)	5.4 (4.0–7.5)
Platelets, X10^9^/L (median; IQR)	235 (159–313)

* See Methods for definitions of inclusion criteria.

** Chronic fever, severe weight loss, chronic diarrhea, moderate weight loss plus CD4<250 cells/µL.

Study subjects were severely immunocompromised with a median CD4 count of 129 (IQR 49–216).

Among patients reporting severe weight loss (>10% of baseline body weight), moderate weight loss (<10% of baseline body weight) and no weight loss, the median BMI was 16.4 (IQR 15.3–17.5), 17.9 (IQR 16.9–19.5) and 18.7 (IQR 16.8–20.0), respectively, (p = 0.001 for differences between all groups).

### Diagnosis of Tuberculosis and Bloodstream Infections

#### Tuberculosis

TB diagnosis was confirmed (IS or blood culture-positive) in 48 and probable (IS smear-positive but culture negative) in 4. IS cultures (46 positive) had a higher yield for MTB than blood (11 positives, of which only 2 were from IS culture-negative participants). IS specimens were smear-positive in 24/46 of the patients with IS culture-positive confirmed MTB. There were an additional 65 possible TB cases based on clinical and/or radiographic features (56% of total TB diagnoses).

#### Bloodstream infections

A total of 46 patients had other BSI, with the most frequent pathogens being NTS (29 patients, 6.2% participants), other gram negative bacteria (9 patients, 1.9%: 5 *Escherichia coli*; 1 *Acinetobacter baumannii*, 1 *Enterobacter sakazakii*, 1 *Citrobacter braakii*, 1 *Shigella dysenteriae*) and 8 *Cryptococcus* (all antigen titre ≥1∶10) of whom 5 also had blood cultures positive for *Cryptococcus neoformans*. A further 4 participants had disseminated NTM disease (blood culture isolate), with 10 other NTM isolates from sputum of undetermined significance. (See Table 2).

**Table 2:** Infections diagnosed among 469 study subjects.

Infection	Number (%)
Confirmed Tuberculosis*	48 (10.2%)
Probable Tuberculosis*	4 (0.9%)
Possible Tuberculosis*	65 (13.9%)
**Total Tuberculosis cases**	**117 (24.9%)**
Nontuberculous mycobacterial disease	4 (0.9%)
Nontyphoidal salmonella species	29 (6.2%)
*E.coli*	5 (1.1%)
Other bacterial pathogens	4 (0.9%)
Cryptococcosis	8 (1.7%)
**Total Bloodstream Infections**	**50 (10.7%)**
**Total All Infections**	**167/469 (35.6%)**

* See Methods for case definitions.

Overall there were 167 patients with a diagnosis of confirmed, probable or possible TB, or a BSI (including NTM). No patient had more than one laboratory-identified pathogen, although 10 possible TB cases were diagnosed among patients with bacteremia (n = 8, of which 7 were NTS) or cryptococcal infection (n = 2). Among the total study cohort there were 62 (13.2%) deaths recorded within 3 months of study enrolment despite the additional diagnostic information provided to clinicians through this study. These deaths occurred in 12/48 confirmed TB cases, 5/65 possible TB cases, 5/29 NTS cases, 2/4 NTM blood culture positive cases, 2/10 NTM IS culture positive cases, 1 cryptococcal infection case and 35/302 subjects with no infection diagnosed.

### Associations between Patient Characteristics and Pathogens

Associations between patient characteristics and laboratory identification of pathogens are shown in Table 3.

**Table 3:** Associations of baseline characteristics with laboratory-diagnosed TB or NTS infections: Multivariate analysis.

All subjects	TB (conf/prob)	NTS
n = 469	n = 52	n = 29
Sex, F	0.67 (0.34–1.33)	0.96 (0.42–2.22)
Age	0.98 (0.95–1.02)	0.98 (0.93–1.02)
Poverty (LQ*)	1.11 (0.51–2.43)	**
Fever, chronic	1.19 (0.56–2.51)	1.43 (0.54–3.76)
Weight loss >10%	1.82 (0.87–3.78)	1.01 (0.42–2.43)
Diarrhea, chronic	0.66 (0.31–1.41)	0.79 (0.29–2.12)
Cough, chronic	1.28 (0.61–2.70)	0.82 (0.31–2.18)
Oral Candidiasis	5.02 (1.48–17.01)	0.99 (0.99–1.01)
Lymphadenopathy	4.27 (1.35–13.56)	0.99 (0.99–1.01)
**Wasting: BMI strata** (kg/m^2^)		
Severe; BMI<16.0	0.97 (0.43–2.23)	0.92 (0.34–2.47)
Moderate; BMI 16.0–16.99	0.82 (0.35–1.96)	**
Mild or no; BMI>17.0	1	**
**CD4 strata** (cells/µL)		
CD4<50	1.01 (0.40–2.53)	4.00 (1.70–9.42)
CD4 50–199	1.09 (0.50–2.38)	**
CD4≥200	1	**
**Anemia** (defined below)		
Severe anemia	3.44 (1.29–9.20)	2.09 (1.00–5.06)
Moderate anemia	1.82 (0.72–4.57)	**
Mild anemia or no anemia	1	**
**Platelets** (X10^9^/L)		
Platelets <50,000	1.03 (0.19–5.53)	**
Platelets 50,000–149,000	0.56 (0.22–1.39)	1.83 (0.72–4.68)
Platelets ≥150,000	1	1
**WBC >8.0** (X10^9^/L)***	0.84 (0.37–1.89)	0.85 (0.27–2.75)

Severe anemia: Hemoglobin <80 g/L (F) & <90 g/L (M).

Moderate anemia: Hemoglobin 80–109 g/L (F) & 90–119 g/L (M).

Mild or no anemia: ≥110 g/L (F) & ≥120 g/L (M).

* LQ  =  Lowest quartile; compared to remainder.

** There were too few cases with these characteristics to include in the regression model.

*** There were also no significant associations with WBC <2.0 X10^9^/L or WBC >10.0 X10^9^/L.

Multivariate analysis of all study subjects showed that only the presence of oral candidiasis, or lymphadenopathy or severe anemia (hemoglobin <80 g/L for women or <90 g/L for men) were independently associated with confirmed or probable TB. CD4 count was not associated with a diagnosis of TB.

Multivariate analysis showed that marked CD4 lymphopenia (CD4<50 cells/µL) and severe anemia (as defined above) were each independently associated with NTS BSI (median CD4 among patients with NTS infection was 46 cells/µL).

Modeling was performed to estimate the sensitivity, specificity and positive and negative likelihood ratios for various combinations of risk factors (fever, oral candidiasis, lymphadenopathy, CD4 lymphopenia, anemia, thrombocytopenia) for confirmed/probable TB and for NTS infection. No combination yielded a positive likelihood ratio of >1.21 for TB or >1.68 for NTS infection (see Table 4).

**Table 4:** Sensitivity & Specificity of Associations with Infections.

Associations with Tuberculosis	Sens	Spec	+LR**	−LR
Chronic fever and/or Anaemia (LQ*) and/or Candidiasis and/or Lymphadenopathy	96%	21%	1.21	0.18
Anaemia (<median) and/or Candidiasis and/or Lymphadenopathy	83%	31%	1.21	0.55
Anaemia (LQ) and/or Candidiasis and/or Lymphadenopathy	64%	47%	1.19	0.78
Anaemia (<median) and/or Chronic Fever	92%	19%	1.15	0.40
**Associations with non-Typhoid Salmonella**				
Chronic fever and/or anaemia (<median) and/or CD4 (<median)	100%	9%	1.1	0
Chronic fever and/or anaemia (LQ) and/or CD4 (LQ)	90%	22%	1.14	0.48
Chronic fever and/or anaemia (LQ) and/or CD4<200	100%	8%	1.09	0
Anaemia (LQ) and/or CD4 (LQ)	72%	57%	1.68	0.49

* LQ  =  Lowest Quartile.

** Positive and Negative Likelihood Ratios (+LR, −LR) are defined in Methods section.

## Discussion

This study shows that TB and NTS were the most commonly identified causes of chronic fever and/or weight loss in smear-negative, HIV-infected outpatients eligible for ART. Serious infections were diagnosed in a third of study participants.

The majority of infections (117/167; 70%) were TB (confirmed, probable and possible cases). A previous study from Malawi of 352 patients classified as smear-negative pulmonary TB found that 39% could be confirmed microbiologically, an equal proportion had clinical and radiological findings compatible with TB but no microbiological confirmation, and that 22% had non-TB diagnoses [29].

Among confirmed and probable TB infections, about half (28/52; 54%) could have been diagnosed with smear microscopy of concentrated IS alone. An additional 24/52 required culture of IS. It has been demonstrated elsewhere that repeated sputum induction can substantially improve the diagnostic yield of this technique [23], [24]. Although 11 cases had positive mycobacterial blood cultures, only 2 required this for diagnosis after smear and culture of IS. Monkongdee et al also found a low yield of mycobacterial blood cultures among an outpatient population [30].

Even enhanced sputum smear microscopy and culture cannot entirely overcome the difficulty we encountered in diagnosing active TB. Overall, 56% of TB cases were possible diagnoses without microbiological evidence to support them. These cases are likely to be a mixture of true TB cases not detected by IS or blood cultures (see Limitations below) and other non-TB diagnoses which may include other undiagnosed infections or HIV-related malignancies.

The high prevalence of TB observed here is consistent with several studies evaluating causes of morbidity and mortality among HIV-infected patients in sub-Saharan Africa [19], [20]. A study from Abidjan, Cote d’Ivoire found a direct correlation between TB and the degree of wasting at autopsy (44% among those with severe wasting) [22]. Cox et al reviewed 20 autopsy series from sub-Saharan Africa and found that TB was responsible for half of the deaths among HIV-infected adults [21].

The surprising finding of our study was the high rate of NTS infection among chronically unwell HIV positive patients, the majority of whom were being treated as out-patients. NTS bloodstream infection accounted for 17% of total cases (including possible TB cases), and half of all blood cultures positive for bacterial, mycobacterial or fungal pathogens. Gordon et al have previously shown that the bacterial load of NTS in blood is low [31] and our consistent use, in this study, of large inoculums of blood may have contributed to the high diagnostic yield.

Invasive NTS are the commonest blood stream bacterial isolates from febrile adult hospital admissions in HIV-prevalent areas of Africa [18], [31]–[33]. However, they have not been described in non-acute admissions/out-patients, raising the possibility that they are a cause of chronic infection in this population group. Recent studies have shown that epidemic invasive NTS in Malawi is caused by a novel host-restricted *Salmonella typhimurium* sequence type (ST313) [34]. Further work is, therefore, required to determine if the NTS isolates we have found in chronic disease are the same genotype as those responsible for infection in acutely unwell, HIV positive patients.

The spectrum of BSI observed in this population of ambulatory chronically ill, HIV-infected patients in a program (non-academic) setting differed from that observed in previous studies of acutely ill, febrile, inpatients among whom *Streptococcus pneumoniae* was an important cause of bacteremia [7], [9], [18], [31], [33], [35]. Immediate inoculation into blood culture bottles, installation onto the blood culture machine an average of 4–6 hours post-collection, and consistently high blood inoculum volumes ensured that contaminants were kept to a minimum (10%) and increased the likelihood of culturing *S. pneumoniae* [36]. Our study enrolment period encompassed wet and dry seasons in Malawi so seasonal variation [8] does not explain the absence of *S. pneumoniae* that we observed. Instead, this may be a true difference in the spectrum of HIV-related BSIs between hospitalised patients and out-patients.

In this study population, microbiological evidence of TB was significantly associated with the presence of oral candidiasis or lymphadenopathy or anemia. Were et al also found an association with lymphadenopathy [15].

Previously published studies have found cough to be associated with the diagnosis of TB [12], [13], [15] but in this study of subjects selected on the basis of negative expectorated sputum smears, as well as fever and/or weight loss, cough was not a useful discriminator.

Hemoglobin measurements are not routinely performed on patients eligible for ART in Malawi. Our systematic determination of Hemoglobin allowed us to examine the utility of Hemoglobin in risk assessments of patients like those recruited into this study. Most patients had some degree of anemia, with median hemoglobin among study subjects being, respectively, 100 g/L (IQR 84–115) for men and 100 g/L (IQR 82–115) for women. Hemoglobin values below the median were significantly associated with both TB, and NTS infection. Saathoff et al found HIV-TB co-infection to be strongly associated with hemoglobin levels and with severe anemia (Hemoglobin <85) [37]. In that study, conducted in Dar es Salaam, Tanzania, the prevalence of severe anemia among co-infected women was 43.4%.

Our analysis shows that although it is possible to define a highly sensitive predictor for the presence of active TB or NTS infection derived from a combination of baseline clinical and/or hematological characteristics, even moderate specificity could only be gained by compromising sensitivity considerably.

### Limitations of the Study

Our use of solid culture media for a single IS culture is likely to have resulted in an underestimation of the true burden of TB and NTM disease. A study by Monkongdee et al evaluated the yield of acid-fast smear and Mycobacterial cultures. In that study a single solid (LJ) culture detected only 47.6% of the infections detected by three liquid cultures (Mycobacterial Growth Indicator Tube). [30] This could have resulted in patients being misclassified as not TB or possible TB when they would have been confirmed TB with fully optimized TB culture; associations are likely to have been diluted through this misclassification.

We did not evaluate patients for malaria. In a hospital-based study of febrile patients in Malawi, 75/233 (32.2%) had chronic fever (>1 month duration); the remainder had acute fever. Among the entire group the prevalence of malaria parasitemia was 4% which was not significantly different than the prevalence among a control group of healthy afebrile adults (7%) [7], [8].

### Conclusions

The high prevalence of TB and BSI in this population of chronically ill patients, with no clear features to allow the different pathogens to be distinguished clinically, has important diagnostic and therapeutic implications. First, our results highlight the importance of TB and the difficulty in excluding TB in this patient group and, secondly, our results also raise the need to consider NTS in outpatient ART clinics. New rapid TB diagnostics now exist with the potential to increase the sensitivity of diagnosis [38], [39] but are unlikely to be widely available at primary care level in resource-limited settings such as Malawi for the foreseeable future. Even if Xpert MTB/RIF were available for testing of smear negative sputum specimens, 28–57% [38], [40] of pulmonary TB infections would be missed unless more than one specimen were tested per patient, with concomitant cost increases. Extrapulmonary TB would not be detected. Sputum induction offers the potential to increase TB case detection by increasing the sensitivity of sputum smear microscopy. Although serum cryptococcal antigen testing could identify most cases of disseminated cryptococcal disease, this infection was diagnosed in less than 2% of this cohort. There are no specific rapid diagnostic tests for the other serious BSI documented here.

Our results suggest a possible role for more aggressive use of presumptive treatment for disseminated infections among high risk patients such as those enrolled in this study. Trials to evaluate empiric TB treatment for high risk HIV-infected patients have been carefully considered and advocated previously [41] and are ongoing. Although autopsy studies provide information about causes of death, they are less helpful in identifying who is at risk of death. It is uncertain to what extent findings from studies of hospitalized patients can be generalized to chronically ill ambulatory patients. In contrast, this prospective study of ambulatory patients enrolled from routine HIV treatment programs has shown that a readily identifiable, high risk group of patients can be defined using any of the inclusion criteria for this study. This bolsters the argument for trials of empiric TB treatment among such patients. Further research should be undertaken to confirm the high prevalence of bloodstream infections found in our study population. Presumptive antimicrobial treatment for these infections, including NTS infection, could also be evaluated in clinical trials particularly for patients with very low CD4 counts. Specific antimicrobial choices for such trials will depend on whether TB is co-treated simultaneously or not, since agents such as fluoroquinolones are active against both NTS and TB.
